# 4098 Health and HIV: Weight status associations with multiple co-morbidities

**DOI:** 10.1017/cts.2020.395

**Published:** 2020-07-29

**Authors:** Kierra Renee Butler, Faye R. Harrell, Jeffrey Robinson, Bridgett Rahim-Williams, Wendy A. Henderson

**Affiliations:** 1 National Institutes of Health

## Abstract

OBJECTIVES/GOALS: Highly Active Antiretroviral Therapy (HAART) is beneficial for managing HIV infection, however the long-term use of HAART may be problematic for healthy weight maintenance. The aim of the study was to investigate the association of race, weight status, and co-morbidities among individuals with HIV. METHODS/STUDY POPULATION: Self-reported data from 283 participants who completed the Symptom Checklist, the Co-Morbidity Questionnaire, and the Sociodemographic Questionnaire were included in the data analyses. Data were analyzed using Latent Class Analysis on JMP 13. Approximately 50% of participants self-identified as Black, 69% as male, and 35% as having AIDS. Participants’ age ranged from 25 to 66 years (mean age = 43.70 years, SD = 8.14). Participants were grouped by race (self- reported Black or non-Black), and then each group was clustered based on the top three most prevalent symptoms. The clusters identified were least symptomatic, weight gain, and weight loss. RESULTS/ANTICIPATED RESULTS: The non-Black weight gain cluster reported a higher incidence of AIDS (70.6% vs 38.2%), nausea (70.6% vs 17.6%), diarrhea (70.6% vs 26.5%), and shortness of breath (58.8% vs 20.6%) compared to the Black weight gain cluster. The Black weight loss cluster reported a higher incidence of cardiovascular symptoms including chest palpitations (42.2% vs 2.7%), chest pain (44.4% vs 8.1%), and shortness of breath (73.3% vs 35.1%) and a higher incidence of all GI symptoms with the most prominent being diarrhea (71.1% vs 48.6%) compared to the non-Black weight loss cluster. DISCUSSION/SIGNIFICANCE OF IMPACT: Future studies supporting these results will assist practitioners to target treatments that may prevent adverse health outcomes for individuals with HIV on HAART. Further studies will also assist with setting standards that allow practitioners to provide personalized care for individuals with HIV on HAART.

